# The role of cellular senescence in metabolic diseases and the potential for senotherapeutic interventions

**DOI:** 10.3389/fcell.2023.1276707

**Published:** 2023-10-06

**Authors:** Huantong Zhang, Han Zhou, Xin Shen, Xingchen Lin, Yuke Zhang, Yiyi Sun, Yi Zhou, Lei Zhang, Dayong Zhang

**Affiliations:** ^1^ School of Medicine, Hangzhou City University, Hangzhou, China; ^2^ School of Economy and Management, Zhejiang Sci-Tech University, Hangzhou, China; ^3^ Taizhou Hospital of Zhejiang Province, Zhejiang University, Taizhou, China

**Keywords:** cellular senescence, metabolic disease, diabetes mellitus, SASP, senolytic

## Abstract

Cellular senescence represents an irreversible state of cell cycle arrest induced by various stimuli strongly associated with aging and several chronic ailments. In recent years, studies have increasingly suggested that the accumulation of senescent cells is an important contributor to the decline of organ metabolism, ultimately resulting in metabolic diseases. Conversely, the elimination of senescent cells can alleviate or postpone the onset and progression of metabolic diseases. Thus, a close relationship between senescent cells and metabolic diseases is found, and targeting senescent cells has emerged as an alternative therapy for the treatment of metabolic diseases. In this review, we summarize the role of cellular senescence in metabolic diseases, explore relevant therapeutic strategies for metabolic diseases by removing senescent cells, and provide new insights into the treatment of metabolic diseases.

## 1 Introduction

Cellular senescence refers to a stable non-proliferative state that cells enter in response to various stresses. This process is implicated in the development of various age-related diseases, including body aging, tumors, and senile dementia (Gorgoulis et al., 2019). Recently, an increasing number of researchers have focused on the relationship between cellular senescence and metabolic disorders. First, key cells involved in metabolic regulation undergo age-related changes. In patients with diabetes, the proportion of aging β-cells in the pancreas increases, and eliminating these cells can effectively prevent the onset and development of diabetes (Thompson et al., 2019). In adipose tissue, aging adipose precursor cells promote insulin resistance in adipose cells (Gao et al., 2020). In addition, aging endothelial cells contribute to the formation of atherosclerotic plaques and increase plaque instability (Bonello-Palot et al., 2014). Second, the secretory phenotype of senescent cells undergoes significant changes, resulting in the production of a variety of pro-inflammatory factors. This phenomenon is referred to as the Senescence-Associated Secretory Phenotype (SASP) (Coppé et al., 2008). As a result, the continued accumulation of senescent cells can cause chronic inflammation. This chronic inflammatory response is considered as an important contributor to metabolic diseases (Wiley and Campisi, 2021). Thus, senescent cells may contribute to the onset and development of metabolic diseases, including diabetes, in various ways. Aging intervention therapies targeting the clearance of senescent cells via senolytics or the modulation of their SASP via senomorphics have increasingly attracted the attention of researchers investigating their potential role in metabolic diseases (Wiley and Campisi, 2021). This paper reviews the relationship between metabolic diseases and cellular senescence and discusses the role of cellular senescence in these disorders, thereby providing new insights into their treatment.

## 2 Definition and characteristics of cellular senescence

### 2.1 Definition of cellular senescence

The concept of cellular senescence describes a cellular state associated with prolonged cell cycle arrest, increased secretory capacity, macromolecular damage, and altered metabolism (Gorgoulis et al., 2019). It occurs as a result of injury or stress in response to endogenous and exogenous stresses, including telomere dysfunction, oncogene activation, and persistent DNA damage. Both pathways, replicative senescence and stress-induced senescence, ultimately activate cytokine-dependent kinase inhibitors such as p53, p21, and p16 ^INK4a^ (Di Micco et al., 2021). In this state, senescent cells can undergo structural changes, including enlarged and flattened morphology, altered plasma membrane composition, lysosomal and mitochondrial accumulation, and significant nuclear expansion (Song et al., 2020). The extrinsic activities of senescent cells are widely associated with SASP activation, which enhances the effects of cell-intrinsic proliferative arrest, which plays a beneficial and positive role in tissue remodeling, wound healing, and tumor suppression during embryonic development (Davan-Wetton et al., 2021; Huang et al., 2022), as well as in the development of a wide range of chronic age-related diseases and organismal senescence (Di Micco et al., 2021).

### 2.2 Characteristics of cellular senescence

#### 2.2.1 Cell cycle arrest

**FIGURE 1:**
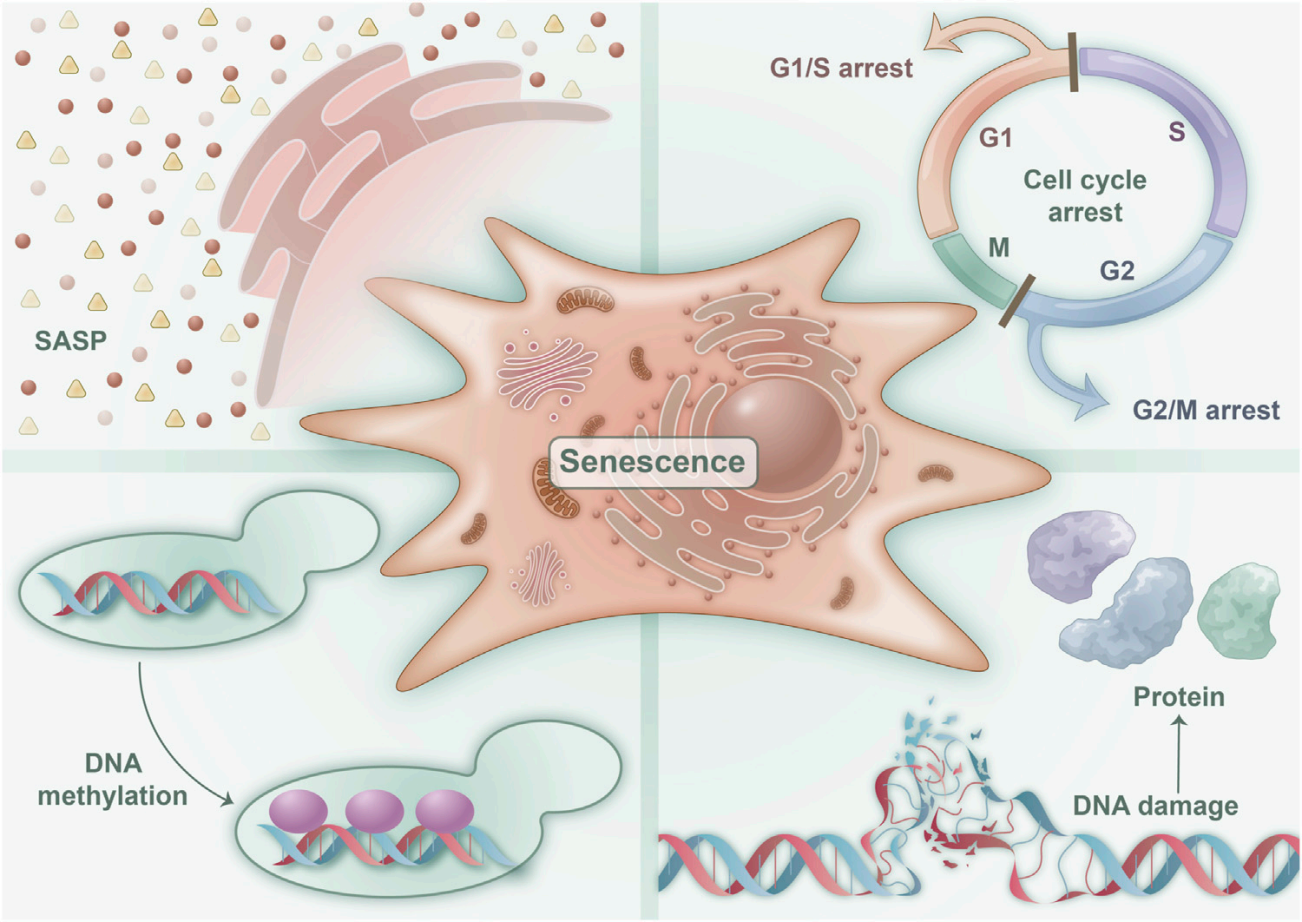
Characteristics of senescent cells: four characteristics are associated with cellular senescence. These are illustrated in the figure and consist of a predominantly irreversible cell cycle block that encourages cellular senescence, SASP, which denotes and mediates various pathophysiological functions of cellular senescence, epigenetic changes in DNA methylation that regulate cellular senescence, and DNA macromolecular damage and proteotoxic damage that initiate cellular senescence.

A notable characteristic of senescent cells is their essentially irreversible cell cycle arrest, which constitutes a vital aspect of tissue repair and tumor suppression. This type of cell cycle arrest differs from quiescence and terminal differentiation, as the former is merely a transient state of cell cycle arrest that can be reversed by suitable stimulation or environmental changes, leading to the resumption of cell proliferation. By contrast, terminal differentiation is a process of cell differentiation that results in the acquisition of specific functions and is accompanied by persistent cell cycle arrest (Figure 1). This process is completely distinct from the mediated pathway of cellular senescence (He and Sharpless, 2017).

Although the cell cycle arrest of senescent cells is primarily considered irreversible, recent studies have revealed that senescent tumor cells can re-enter the cell cycle under the influence of certain specific factors (Saleh et al., 2019). Moreover, hyperinsulinemia induces senescence in fat cells (Li et al., 2021) and nerve cells (Chow et al., 2019), yet it can restart the cell cycle by upregulating cell cycle–promoting factors. Despite the inability of these cells to complete division ultimately, the cell volume increases, and more inflammatory factors are secreted, indicating that the regulation of the cell cycle in senescent cells is intricate and mediates cellular senescence promoted by metabolic abnormalities.

#### 2.2.2 SASP

Senescent cells exhibit a distinctive secretory activity and possess the ability to release a vast array of factors, including proinflammatory cytokines, chemokines, growth regulators, angiogenic factors, and matrix metalloproteinase (Figure 1). This phenomenon is commonly referred to as SASP, which stands for senescence-messaging secretome. SASP represents the cardinal trait of senescent cells, and it mediates their diverse pathophysiological functions. Notably, SASP triggers the senescence of neighboring cells via the autocrine and paracrine pathways, amplifying and broadening the scope and extent of cellular senescence (Su et al., 2019). The accumulation of senescent cells and SASP can precipitate a plethora of age-related ailments. However, notably, acute SASP can impede tumor growth and constrain fibrosis (Birch and Gil, 2020). Consequently, SASP serves as a critical feature of senescent cells, controlling their regulatory functions in physiology and various disease states (Gorgoulis et al., 2019).

#### 2.2.3 Epigenetic changes

Epigenetics refers to the phenomenon where the DNA sequence remains unaltered, but heritable changes arise in gene expression. Numerous studies on aging demonstrate that epigenetic changes occur during cellular senescence, primarily manifested through changes in DNA methylation, histone modification, chromatin remodeling and SASP (Crouch et al., 2022) (Figure 1). Methylation and acetylation are two important epigenetic modifications that play a role in cellular senescence. In senescent cells, there is a global loss of DNA methylation and an imbalance of activating and repressive histone modifications (Sen et al., 2016). Specifically, a loss of histones occurs concurrently and an increase in repressive modifications, such as H3K9me3 and H3K27me3, associated with heterochromatin formation and gene silencing, while concomitantly experiencing a decrease in activating modifications, such as H3K4me3 and H3K9ac, which are linked to euchromatin formation and gene activation (Jambhekar et al., 2019). These changes in histone modifications are thought to contribute to the transcriptional changes observed in senescent cells. Environmental factors, such as dietary restriction and exercise, can regulate cellular senescence by altering epigenetic inheritance through metabolism and other means (Hernandez-Saavedra et al., 2019).

#### 2.2.4 Macromolecular damages

The primary molecular characteristic of replicative cell senescence is telomere shortening. Telomere shortening occurs because of the asymmetric replication of the two ends of DNA during continuous passage, leading to a small loss of DNA telomere repeats. In addition, telomere shortening during proliferation results in telomere DNA instability and telomere uncapping, which activates DNA damage repair and triggers telomere dysfunction, leading to cell cycle arrest (Di Micco et al., 2021) (Figure 1). Moreover, protein toxicity is a marker of cell senescence (Ishikawa and Ishikawa, 2020). Reactive oxygen species (ROS) in senescent cells cause protein damage by oxidizing methionine and cysteine residuals, altering protein folding and function, and leading to protein damage in senescent cells. Studies indicate that lipids are critical for cell membrane integrity, energy production, and signal transduction (Ademowo et al., 2017). Cellular senescence also results in lipid damage. Many senile diseases, such as abnormal lipid metabolism in metabolic syndrome (MetS), have been associated with extensive lipid damage in senile cells, leading to an increase in senescent cells and intracellular lipid damage (Ademowo et al., 2017).

## 3 Metabolic diseases and cellular senescence

**FIGURE 2:**
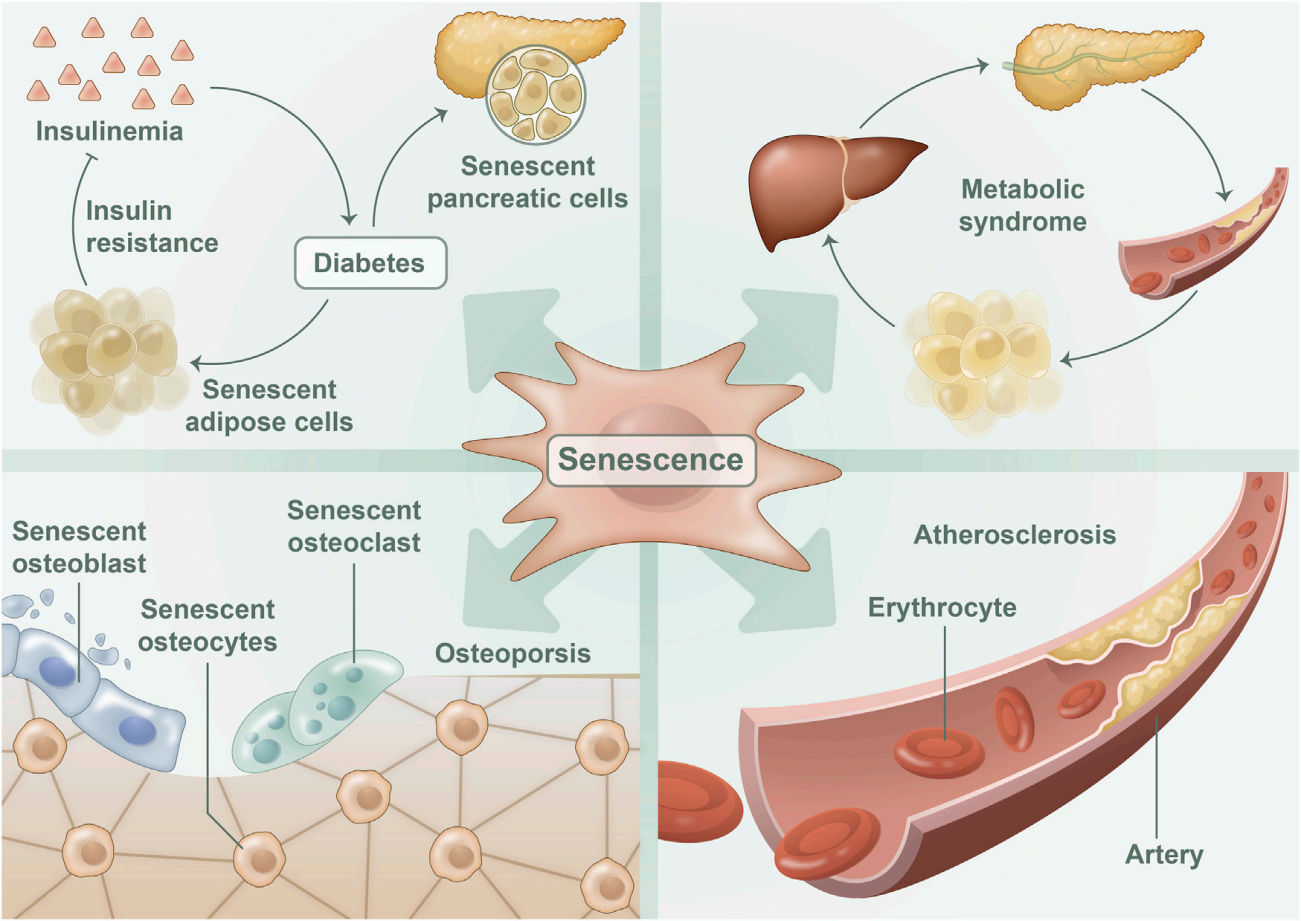
Cellular senescence in metabolic diseases: The process of cellular senescence has been implicated in the development of various metabolic diseases. Senescent adipocytes and islet beta cells perform a significant function in the onset of diabetes, whereas senescent adipocytes also contribute to the development of metabolic syndrome. In osteoporosis, one can observe the presence of senescent osteoblasts, osteocytes, and osteoclasts. Moreover, senescent endothelial cells were detected in atherosclerotic disease.

Research on aging indicates that cellular senescence is a critical risk factor for a variety of metabolic diseases, including MetS (Dominguez and Barbagallo, 2016), diabetes (Palmer et al., 2019a), metabolic cardiovascular diseases (Evangelou et al., 2023), and osteoporosis (Polyzos et al., 2021) (Figure 2). Cellular senescence can disrupt organ homeostasis and promote metabolic disease through mechanisms such as promoting systemic inflammation, oxidative stress, and metabolic dysregulation (Cai et al., 2022). Conversely, delaying or diminishing cellular senescence may mitigate metabolic diseases to some degree (Wiley and Campisi, 2021). These findings underscore the strong link between cellular senescence and metabolic diseases, as cellular senescence plays both a direct and indirect role in their initiation and serves as a critical regulator in metabolic diseases.

### 3.1 Diabetes mellitus (DM)

The incidence of DM, particularly type 2 diabetes mellitus (T2DM), increases with age. Prior investigations have demonstrated the close association between DM and cellular senescence (Palmer et al., 2019a). First, T2DM may stimulate cell senescence, and the quantity of senescent cells in adipose tissue of T2DM mouse models is remarkably greater than that of the control group (Ahima, 2009). In addition, adipose tissue senescence can diminish the expansion potential of adipose tissue, thereby resulting in insulin resistance (Hammarstedt et al., 2018). These findings suggest that adipocyte senescence plays a pivotal role in mediating the development of DM. Second, the islet β-cells themselves experience aging-related changes. The expression of the age-related gene p16^INK4a^ in β-cells also increases with age, which impairs the insulin secretion function of aging β-cells. Furthermore, cellular senescence can directly influence the function of islet β-cells and produce many inflammatory factors, leading to chronic inflammation of pancreatic tissue, markedly contributing to the onset and progression of T2DM (Murakami et al., 2022).

#### 3.1.1 Adipocyte senescence

Several studies have demonstrated that obesity can trigger cellular senescence, and senescent cells show a remarkable increase in adipose tissue in individuals with obesity, thereby leading to the development of T2DM. T. Minamino et al. observed that adipose cells in KK-Ay mice, who consumed excessive calories, exhibited augmented senescence-associated β-galactosidase (SA-β-Gal) activity, elevated p16 ^INK4a^ and EGFP expression, and increased SASP factor (Minamino et al., 2009). Furthermore, senescence occurred in these adipose cells before insulin resistance, inflammation, and glucose tolerance in adipose tissue, indicating a causal relationship between excessive energy intake and adipose cell senescence (Minamino et al., 2009).

Another study revealed that the expression of p53 in adipose cells is glucose reactive and a critical mediating factor in high-sugar-induced adipose cell senescence (Krstic et al., 2018). The expression of p53 must be downregulated for adipose mesenchymal stem cells (ADMSCs) to develop into insulin-responsive adipocytes. However, in T2DM, the activity of p53 in ADMSCs is heightened, leading to an increased level of cellular senescence (Minamino et al., 2009).

Moreover, excessive caloric intake not only induces senescence in adipose tissue but also accelerates cellular senescence in other tissues of the body via senescent adipose tissue. This phenomenon promotes islet β-cell senescence, thereby increasing the risk of developing T2DM (Burton and Faragher, 2018). In mouse models, senescent adipocytes have been found to trigger T2DM by causing insulin resistance (Petersen and Shulman, 2018), adipose tissue inflammation (Palmer et al., 2019b), and immune cell attraction (Palmer et al., 2019b). Activating p53 in senescent cells decreases glucose transport and increases lipolysis, which results in a further reduction of insulin-responsive adipocytes, leading to inflammation and insulin resistance (Krstic et al., 2018). Consequently, senescent cells in mice with T2DM can also lead to adipose tissue dysfunction, which further triggers systemic metabolic changes such as insulin resistance, ectopic lipid accumulation, and chronic inflammation, thereby exacerbating the complications of T2DM (Spinelli et al., 2020). In mice with diet-induced obesity, senescent cells can attract macrophages into adipose tissue and worsen T2DM (Palmer et al., 2019b). Therefore, adipose tissue cell senescence can contribute to the development of T2DM through several mechanisms, including insulin resistance and inflammatory response.

#### 3.1.2 Islet β-cell senescence

Senescence of islet β-cells has a remarkable impact on the pathogenesis of T2DM. Islet β-cells isolated from older mice and those with T2DM have increased the activity of SA-β-Gal (Aguayo-Mazzucato et al., 2019). Furthermore, T2DM model mice have shown a higher expression level of insulin-like growth factor 1 receptor (IGF-1R) in their β-cells compared with control mice, indicating a close relationship between pancreatic β-cell senescence and T2DM (Yan et al., 2022). Studies have also revealed that insulin resistance induction can lead to augmented expression of pancreatic β-cell senescent markers, including a high expression level of SA-β-Gal, p16 ^INK4a^, p53^BP1^, and IGF-1R. As the secretion of basic insulin increases, pancreatic function impairment continuously hastens the senescence of pancreatic β-cells, ultimately resulting in T2DM (Aguayo-Mazzucato et al., 2017).

**FIGURE 3:**
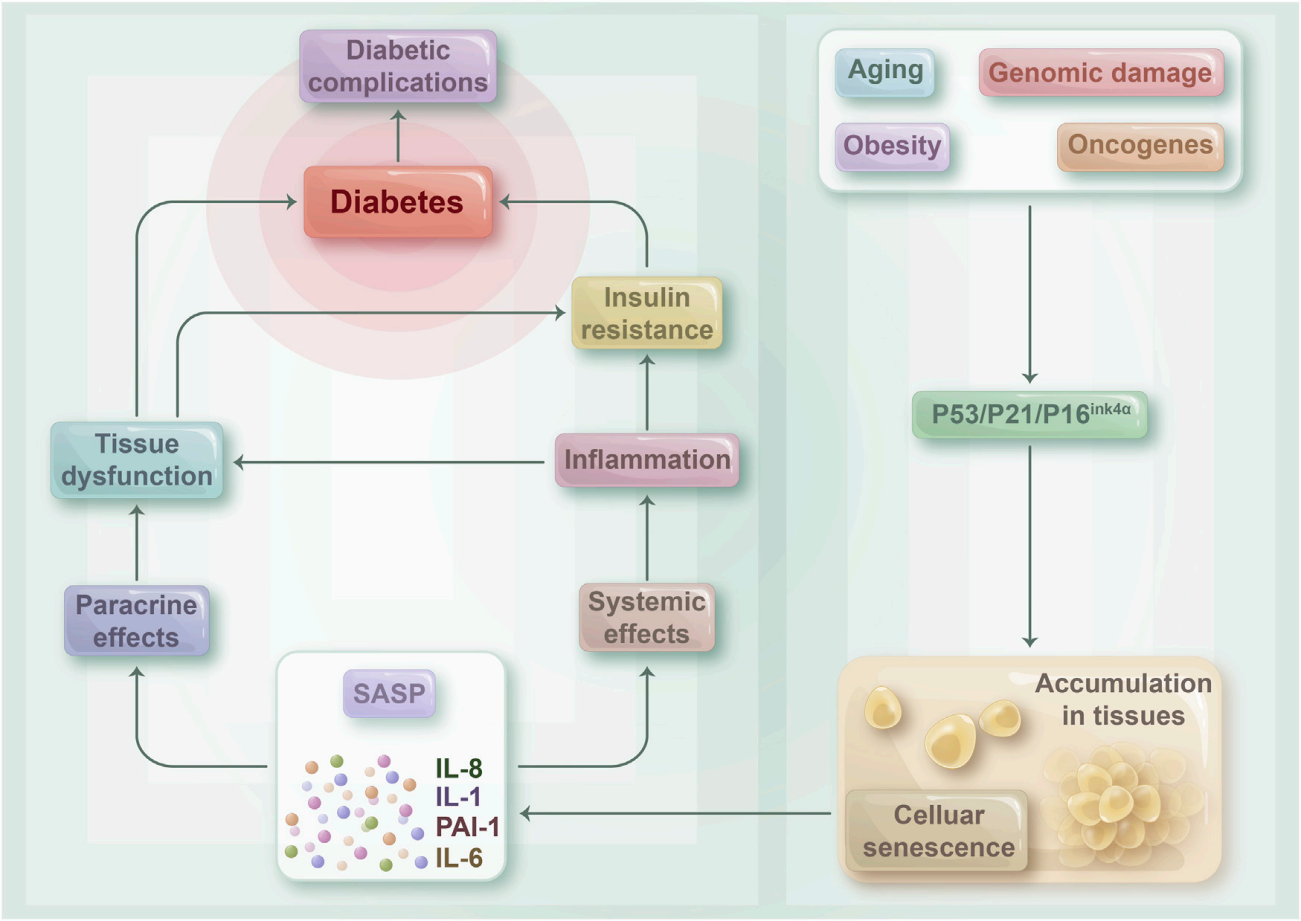
The relationship between cellular senescence and diabetes: Senescent cells may play a crucial role in contributing to insulin resistance and the development of diabetes mellitus, as well as its associated complications. As the body undergoes the aging process, becomes obese, or experiences various disease states, senescent cells tend to accumulate in tissues throughout the body. This accumulation triggers the release of a range of pro-inflammatory cytokines, chemokines, and growth factors known as SASPs, which can act both locally and systemically. SASPs can target cells within tissues via paracrine mechanisms, which can result in tissue dysfunction and damage. Such damage can ultimately lead to the development of diabetes and its associated complications. Moreover, SASP can circulate throughout the body and contribute to a tense inflammatory state, thus playing a role in the development of insulin resistance.

Studies on aging have revealed that senescent islet β-cells exhibit reduced insulin secretion ability and less sensitivity to high sugar stimulation (Tudurí et al., 2022). Insulin resistance is the pivotal link between cell senescence and the development of T2DM, which also directly impacts the functioning of pancreatic β-cells. In 2017, Aguayo Mazzucato’s experiments demonstrated that insulin resistance in insulin-resistant mouse models could increase the expression level of senescent markers of pancreatic β-cells, such as p16^INK4a^, IGF-1R, and BAMBI, thereby accelerating the senescence of pancreatic β-cells (Aguayo-Mazzucato et al., 2017). As mentioned previously, a high-calorie diet can promote fat cell senescence and lead to insulin resistance (Krstic et al., 2018). However, insulin resistance can further induce the senescence of pancreatic β-cells, thereby causing T2DM (Murakami et al., 2022). Therefore, β-cell senescence and insulin resistance exhibit a causal relationship and promote each other, thereby aggravating the development of T2DM (Figure 3).

Bisphenol A (BPA), which is considered as a prevalent endocrine disruptor, can cause insulin resistance and promote the progression of T2DM. In 2019, Avinash Soundararajan et al. discovered that the transcription level of pancreatic β-cell senescence markers, such as p15, p53, Rb1, and SA-β-Gal, increased when metabolic stress was combined with BPA. This finding indicates that BPA can promote pancreatic β-cell senescence (Soundararajan et al., 2019). In addition, they used zebrafish embryos as models to verify whether the group exposed to hyperglycemia and BPA showed higher levels of islet β-cell apoptosis and senescence compared with the control group (Soundararajan et al., 2019). These studies suggest that pancreatic β-cell senescence plays an important role in BPA’s promotion of T2DM.

### 3.2 MetS

MetS is a group of syndromes characterized by a combination of metabolically related risk factors, which include central obesity, hyperglycemia characterized by insulin resistance or reduced glucose tolerance, hypertriglyceridemia, low levels of HDL and cholesterol, and hypertension. MetS is widely considered as a major risk factor for diseases such as diabetes and cardiovascular and cerebrovascular diseases. Without intervention, around 50% of patients with MetS will develop diabetes (Alberti et al., 2005). Recently, MetS has been found to be closely linked to cellular aging, with sustained high-calorie intake being identified as an important cause of MetS. Our research group (Zhang et al., 2017) and other research groups (Ogrodnik et al., 2019) have demonstrated through experiments that high-calorie intake can promote senescent changes in adult cells and stem cells. As individuals age and develop MetS, the level of oxidative stress in their bodies and cells increases, and ROS generated by oxidative stress can induce macromolecular damage. The increase in intracellular ROS levels is the primary mechanism underlying adipocyte and macrophage senescence (Nunn et al., 2009). A more descriptive term for MetS is “lifestyle-induced metabolic inflexibilities and accelerated senescence syndrome.” Studies have suggested that insulin resistance is the primary pathogenic mechanism of first-level metabolic changes in patients with MetS, whereas chronic low-level inflammation and oxidative stress are second-level changes. Furthermore, the release of a large number of inflammatory factors by senescent cells is considered as an important mechanism of cellular senescence involved in MetS (Fahed et al., 2022). Therefore, MetS and cellular senescence are mutually reinforcing, with cellular senescence aggravating the occurrence and development of MetS and being a cellular manifestation of progressive MetS.

Studies have demonstrated that various age-related phenomena in the body, such as obesity, inflammation, hypertension, hypothalamic–adrenal pituitary activity, and insulin resistance, can increase the prevalence of MetS (Fahed et al., 2022). In turn, MetS is further exacerbated by cellular senescence, insulin resistance, and cardiovascular disease.

Insulin resistance is a crucial factor in the development of MetS. The insulin receptor substrate (IRS) protein plays an important role in insulin–insulin receptor (IR) binding. Upon insulin stimulation, the IR and IRS are phosphorylated by activated insulin receptor kinases, which leads to the activation of insulin signaling factors in the plasma membrane and internal membrane cisterna. These signaling factors transmit insulin signals and produce typical insulin-induced metabolic effects, such as increased glucose uptake. However, during cellular senescence, the level of intracellular oxidative stress is heightened, activating phosphorylation cascades, such as mitogen-activated protein kinase cascades (Yaribeygi et al., 2019). This activation leads to the increased Ser/Thr phosphorylation of IRS molecules, releasing modified IRS molecules from the internal membrane pool, promoting protein degradation, and ultimately resulting in further insulin resistance. Mitochondrial function improvement and ROS level reduction through gene editing decreased the expression level of senescent markers p16 ^INK4a^ and p21, and the condition of gene-edited mice with MetS was remarkably improved (Tavallaie et al., 2020). Elevated oxidative stress levels in cellular senescence enhance insulin resistance and other effects, which caused the inability of insulin to achieve normal metabolic effects, ultimately leading to further MetS deterioration.

MetS can also induce senescence in mesenchymal stem cells (MSC) and affect the content of micro-RNA (miRNA) and aging-related miRNA in MSC-derived extracellular vesicles. In 2018, Meng et al. (2018) found that the MetS model had seven upregulated and three downregulated miRNAs in adipose tissue–derived MSC, which regulated 35 age-related genes and induced oxidative stress by increasing a SASP factor and the senescence regulatory factor integrin-β1, thereby worsening the occurrence and development of MetS.

### 3.3 Osteoporosis

**FIGURE 4:**
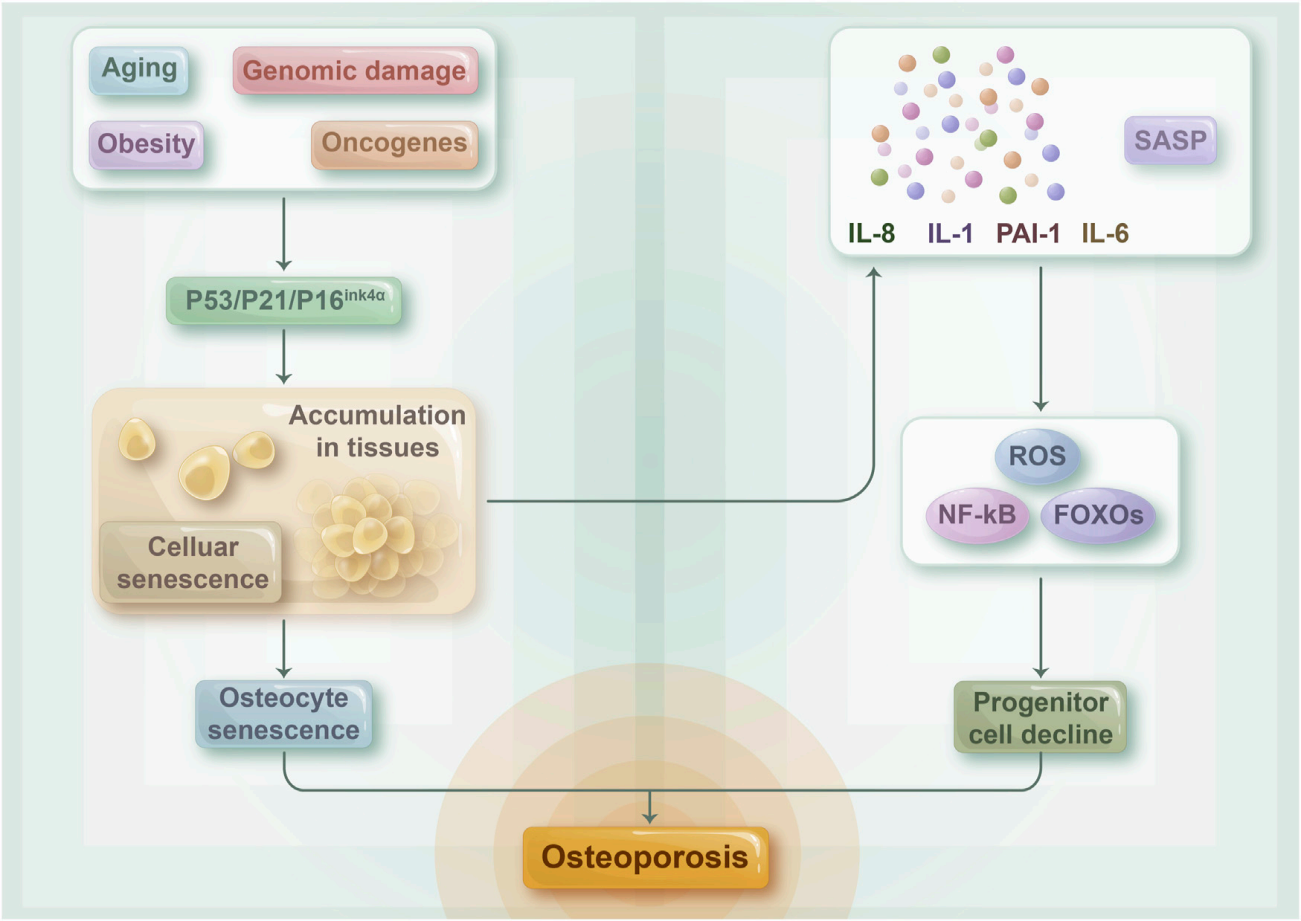
The relationship between cellular senescence and osteoporosis: The composition of senescent osteocytes and their SASP may play a role in the progression of age-related osteoporosis. As the aging process advances, numerous cells in the bone microenvironment undergo senescence and accumulate, while senescent osteoblasts are principally responsible for the development of SASP. This, in turn, activates NF-kB, ROS, and FOXOs, indicating a decline in the number of osteoprogenitor cells. Collectively, these factors contribute to the connection between cellular senescence and osteoporosis.

Osteoporosis is prevalent among metabolic bone diseases and is a significant ailment associated with aging. In general, estrogen-deficient postmenopausal women experience severe bone loss or even fractures caused by osteoporosis. Cellular senescence has been identified as a key factor in bone loss, while the development of osteoporosis is triggered by a combination of estrogen, inflammatory factors, and cellular senescence (Farr and Khosla, 2019) (Figure 4). Evidence suggests that estrogen deficiency leads to osteoporosis by influencing inflammation levels (Weitzmann and Pacifici, 2006; Mohamad et al., 2020). Apart from its capacity to suppress the inflammatory response, estrogen is closely linked to cellular senescence. Studies have shown that estrogen deficiency causes oxidative stress and osteoblast senescence in a mouse model of osteoporosis. Cells exhibits senescence symptoms such as delayed growth rate and impaired proliferation, whereas senescent osteoblasts can trigger an inflammatory microenvironment via SASP (Kassem and Marie, 2011; Geng et al., 2019). Joshua N et al. proved that senescent cells play a causative role in mediating age-related bone loss under aging conditions. Their experiments demonstrated that senescent osteoblasts play a crucial role in SASP production, and the expression level of pro-inflammatory chemokines, cytokines, and extracellular matrix degradation proteins, which drive age-related bone loss, was upregulated with the increase of SASP levels (Farr et al., 2019). The inflammatory microenvironment can directly affect osteoblast and osteoclast physiological functions, leading to osteoporosis (Peng et al., 2022). Additional experiments have shown that older mice have increased levels of osteoblast senescence, which causes a senescent phenotype in the bone marrow lumen’s myeloid cells via SASP with age. This phenomenon ultimately results in a toxic inflammatory microenvironment, which affects a wide range of adjacent cells, including B cells, T cells, osteoblasts, and osteogenic progenitor cells, and increases the expression level of p16 ^INK4a^, a key mediator of cellular senescence, thereby resulting in osteoporosis (Farr et al., 2016b). Therefore, estrogen, inflammation, and cellular senescence are closely linked to osteoporosis.

Aged (24 months old) mice display notable senescent traits in their bone tissue, such as osteoblasts, osteocytes, and bone marrow mesenchymal stem cells, in addition to a decline in bone mass when compared with young (6 months old) mice (Farr et al., 2016a). Furthermore, a study on a senescence-accelerated model demonstrated that an upsurge in senescent osteoblasts decreased the bone formation capacity, culminating in osteoporosis in model mice (Wang et al., 2012). These findings imply that cellular senescence plays a role in osteoporosis development and is a crucial underlying cause of this disease.

### 3.4 Metabolic cardiovascular disease

Cardiovascular maladies frequently coexist with obesity, diabetes, dyslipidemia, and other metabolic anomalies. Clinically, metabolic cardiovascular diseases are closely related to metabolic abnormalities. It is a clinical syndrome wherein atherosclerosis is the main pathophysiological change, and cardiovascular and cerebrovascular events are the primary outcomes. The intervention of metabolic disorders can improve the prognosis (Stefan, 2020; Herman and Birnbaum, 2021).

In metabolic cardiovascular disease, aging is considered as a crucial independent risk factor for pathological manifestations such as atherosclerosis. Senescent endothelial cells are often detected in atherosclerotic plaques of elderly patients, thereby indicating that endothelial cell senescence might be a possible factor in the occurrence and development of atherosclerosis (Bonello-Palot et al., 2014). Research has demonstrated that the senescence of endothelial cells can contribute to the primary pathological indications of metabolic cardiovascular illnesses such as atherosclerosis through several mechanisms. The initial alterations and significant aspects of metabolic cardiovascular illnesses such as atherosclerosis are impaired endothelial function and increased inflammatory factors resulting from endothelial cell senescence, which are evidenced by chronic inflammation, oxidative stress, and decreased nitric oxide (NO) bioavailability closely associated with cellular senescence. All of these factors contribute to cardiovascular harm (Shakeri et al., 2018). For example, NO released by normal endothelial cells can prevent platelet aggregation, while reduced activity of endothelial nitric oxide synthase and decreased NO production are discovered in senescent endothelial cells (Lee et al., 2017). Normal endothelial cells produce NO that can impede apoptosis, such as inhibiting caspases, which are crucial factors in the apoptotic cascade; however, aging endothelial cells have a reduced NO bioavailability, and such cells cannot hinder endothelial apoptosis induced by diverse stimuli, leading to impaired endothelial function. In addition, the SASP from senescent endothelial cells can incite widespread inflammation in endothelial tissue, promoting atherosclerosis (Sun et al., 2022). Furthermore, extracellular vesicles generated by normal endothelial cells sustain intravascular homeostasis, whereas microvesicles produced by senescent endothelial cells have been found to stimulate the calcification of vascular smooth muscle cells, thereby contributing to vascular injury (Alique et al., 2017).

Other studies have displayed that the expression level of pro-inflammatory cytokines such as IL-1β, IL-6, TNF-α, and interferon γ is increased in the elastic arteries of the elderly (Wang et al., 2007). Simultaneously, increased levels of various adhesion molecules and decreased levels of thromboregulatory proteins have been found in senescent vascular endothelial cells, which promote atherosclerosis (Minamino and Komuro, 2007). Hence, cellular senescence not only causes metabolic cardiovascular diseases but also provides a novel entry point for the treatment of metabolic cardiovascular diseases.

## 4 Therapeutic potentials of targeted removal of senescent cells

As previously mentioned, cellular senescence in metabolic disorders plays a pivotal role in their development from various perspectives. Thus, investigating new therapeutic strategies that target the elimination of senescent cells is necessary. Compared with conventional treatments, the targeting of senescent cells may have beneficial therapeutic effects by reducing the abundance of senescent cells (Fafian-Labora et al., 2020). This approach can prevent the early onset of metabolic diseases and effectively manage metabolic diseases, such as T2DM. Consequently, the interventional approach of selectively removing senescent cells is considered as a valuable therapeutic option for metabolic disorders.

### 4.1 Targeted therapies to remove senescent cells

As research into aging continues to progress, numerous compounds have been developed, which target the elimination of senescent cells (Senolytics) or the inhibition of SASP phenotypes (Senomorphics) in response to the apoptotic resistance and other characteristics of senescent cells, along with targeted antibodies, cell therapy, cytokine induction, and other therapeutic approaches. Removing senescent cells, breaking the vicious cycle of inflammation and cellular senescence, and restoring the physiological function of patients with metabolic diseases have shown great therapeutic potential.

Recent experiments have demonstrated that senolytics such as quercetin, dasatinib, and laccasein can delay or even reverse the aging process, improve metabolic profiles. Quercetin can act on several therapeutic targets in diabetes, including inhibiting intestinal glucose absorption, improving insulin secretion, enhancing insulin action, improving glucose utilization in peripheral tissues (Dhanya, 2022). In natural aging models in mice treated with laccasein, SASP factors were significantly reduced, oxidative stress decreased, tissue homeostasis restored, and the maximum median life span of mice increased (Yousefzadeh et al., 2018). In studies involving adipose tissue, treatment with a senolytic combination of dasatinib with quercetin (Dasatinib plus Quercetin, D+Q) resulted in decreased expression of p21 and p16 ^INK4a^ in adipose tissue, significantly reduced expression of pro-inflammatory cytokines, and improved glucose tolerance and insulin resistance in mice with obesity (Palmer et al., 2019a). Bcl-2 inhibitors reduced the level of islet inflammation by selectively eliminating senescent β cells with high Bcl-2 expression, thereby preventing or delaying the onset of T1DM (Thompson et al., 2019). In animal experiments in osteoporosis, either D+Q treatment and genetically encoded elimination of senescent cells or the use of JAK inhibitors suppressed the production of a pro-inflammatory secretome of senescent cells, remarkably improving the osteoblast microenvironment in mice, increasing bone mass and strength, and obtaining better bone microarchitecture (Farr et al., 2017). In aged or atherosclerotic mouse models, treatment with D+Q reduced the number of senescent cells in the middle aortic layer and improved vasodilatory function (Roos et al., 2016). These studies further confirm the causal relationship between senescent cells and metabolic diseases such as osteoporosis and DM and suggest that the removal of senescent cells may not only delay the development of metabolic diseases but also produce positive therapeutic effects.

Additionally, the development of novel senolytics, targeted anti-aging drugs and the pursuit of regenerative therapies through metabolic intervention are all endeavors that deserve significant attention. A study using CAR-T cells as senolytics, targeting the specific protein urokinase-type fibrinogen activator receptor on the surface of senescent cells, was effective in removing senescent cells *in vitro* and *in vivo*, prolonging survival and restoring tissue homeostasis in model mice (Amor et al., 2020). Other recent studies have used glutaminase 1 inhibitors as senolytics to inhibit glutaminolysis and induce acidosis and death of senescent cells, with improved physiological function and motility in the experimental group of aged mice (Johmura et al., 2021). These studies suggest the potential of senolytics for greater application in the treatment of metabolic diseases. Moreover, antibody-drug conjugates targeting the senescent surface group marker B2M selectively killed senescent cells without causing toxicity to proliferating cells (Poblocka et al., 2021). This finding demonstrates the feasibility of antibody-based targeted anti-aging drugs and provides a new avenue for the development of novel drugs. And in investigating the importance of metabolic intervention in tissue repair and regeneration, a recent study identified metabolites, including uridine, that can rejuvenate aged stem cells and promote tissue regeneration through a combination of metabolomics analysis and small molecule screening (Liu et al., 2022), potentially paving the way for the development of new regenerative therapies to combat cellular senescence.

Although the targeted elimination of senescent cells has demonstrated greater potential for treating diseases, recent research has revealed that the therapeutic effect is also directly affected by the type of cells selected for senescent cell removal, as well as the rate and timing of removal. In studies conducted on knockout mice, the rapid removal of senescent hepatic sinusoidal endothelial cells that expressed high levels of p16^Ink4a^ resulted in the ineffective replacement of these cells, thereby disrupting the blood tissue barrier and triggering fibrosis in the liver and perivascular tissues (Grosse et al., 2020). Furthermore, senescent cells increased in the liver of rats during hemorrhagic shock, where the senescent cells played a positive role in injury repair by producing the SASP, and their removal through the D+Q method resulted in the rapid death of the rats (Chu et al., 2020). At present, fewer studies have been conducted to understand the molecular mechanism of senolytics and senomorphics, as well as their therapeutic potential, and our knowledge of their therapeutic strategies remains limited. Nonetheless, concentrating on the therapeutic value of senolytics in metabolic diseases and conducting clinical trials are necessary. Furthermore, senolytics, which eliminate senescent cells, will be a novel drug for the treatment of metabolic diseases.

### 4.2 Therapeutic role of senescent cell removal in the treatment of metabolic diseases

In metabolic disorders, cellular senescence is a major contributor to their development by inducing inflammatory responses and diminishing cellular functions. The SASP is a significant factor in the contribution of cellular senescence to metabolic diseases. Senescent cells continuously release cellular inflammatory factors, including IL-1, IL-6, and IL-12, and stimulate the conversion of pro-inflammatory M1 macrophage polarization, thereby activating the inflammatory response during the early stages of injury (Lau et al., 2019; Zhang et al., 2019). In turn, the persistent inflammatory response is a key etiology of metabolic diseases such as atherosclerosis, DM, and osteoarthritis.

The expression level of IL-1α, IL-6, CXCL1, and TNF-α was reduced; the locomotor and coordination abilities of experimental mice were enhanced, and metabolic disorders were improved after the targeted removal of senescent cells using the β-galactosidase-targeted prodrug SSK1 in different tissues (Cai et al., 2020). The abovementioned study illustrates that the targeted removal of senescent cells can alleviate the inflammatory microenvironment, which leads to therapeutic effects on metabolic diseases.

Moreover, senescent cells not only produce SASP to trigger tissue dysfunction, but also accelerate the rate of senescence in surrounding cells and expand the scope of senescence in surrounding tissues, leading to a series of changes such as persistent DNA damage response in pancreatic β-cells, vascular endothelial cells (Zhao et al., 2020), senescence-associated mitochondrial dysfunction (Miwa et al., 2022) and ultimately promoting metabolic disorders such as T2DM, MetS, osteoporosis, and atherosclerotic cardiovascular diseases. Therefore, the elimination of senescent cells directly protects the key cells of metabolic regulation, protects the metabolic function of the body, and prevents or treats metabolic disorders (Table 1).

**TABLE 1:** The interventional effects of senotherapy in metabolic diseases.

	Types/Complications	Agent	Main effects	Cellular senescence-related signalling pathways	Targeting senescent cell types
Diabetes	T2DM	Quercetin	Reduces blood glucose concentration and regulates key signalling pathways to reduce ROS damage Dhanya (2022)	Bcl-2 family, p53/p21/serpine, and PI3K/AKT	Pancreatic β-cells
	T1DM	Navitoclax (ABT-263)	Selectively binds to Bcl-2, Bcl-XL, and Bcl-w, eliminates senescent beta cells with high expression of Bcl-2, and reduces the level of islet inflammationThompson et al. (2019); Sreekumar et al. (2020)	Bcl-2 family (Bcl-2, Bcl-xL, Bcl-w)	Pancreatic β-cell HUVECs
	DKD	D + Q	Downregulation of senescence-associated cells, reduction of SASP factors in the blood, and reduction of senescent cell load in adipose tissue Hickson et al. (2019)	Dependent receptor/Src kinase/tyrosine kinase, Bcl-2 family, p53/p21	Adipocytes and adipose progenitor cells
	PDR	UBX-1325	enhanced the effects of anti-VEGF drugs, downregulated SASP and general aging, and also improved the OIR and STZ-induced retinal vasculature Crespo-Garcia et al. (2021); Hassan and Bhatwadekar (2022)	BCL-xL inhibitor	HUVECs, monocytes, retinal cells
	PDR	UBX1967	activation of cystein-3 and other intrinsic pathways that trigger apoptosis. Improved the retinal vascular system, allowing the reorganization of vascular units Crespo-Garcia et al. (2021); Hassan and Bhatwadekar (2022)	BCL-xL inhibitor	HUVECs, retinal cells
Atherosclerosis	\	Navitoclax (ABT-263)	ABT-263 eliminated dysfunctional senescent cells from atherosclerotic plaques to delay atherosclerosis, respectively Sreekumar et al. (2020)	Bcl-2 family (Bcl-2, Bcl-Xl, Bcl-w)	HUVECs
	\	D + Q	It reduces the number of senescent cells in the middle aorta and improves vasodilatory function Roos et al. (2016)	Dependent receptor/Src kinase/tyrosinase, Bcl-2 family, p53/p21/PI3K/AKT, etc	Aortic cells
Osteoporosis	\	D + Q	Elimination of senescent cells with higher bone volume and strength and better bone microarchitecture Farr et al. (2017)	Dependent receptor/Src kinase/tyrosine kinase, Bcl-2 family, p53/p21, etc	Osteoclast
	\	Jak Inhibitors	Inhibit the pro-inflammatory secretion group of senescent cells, significantly improve the microenvironment of mouse bone cells, and increase the bone volume and strength of mouse bones Farr et al. (2017)	Selective inhibition of JAK kinase, blocking JAK/STAT signaling pathway	Osteoblast/osteoclast\CFU-M
	\	Lacrimalin (Flavonoid)	Lacquer inhibits osteoclast differentiation by suppressing the NFATc1 signaling pathway while stimulating osteoblast differentiation by increasing RUNX2 expression Molagoda et al. (2021)	PI3K/AKT	Osteoblasts/osteoclasts

DKD, diabetic nephropathy; PDR, diabetic retinopathy; AMD, age-related macular degeneration; D, dasatinib; Q, Quercetin.

## 5 Discussion

A complex and dynamic interplay exists between cellular senescence and metabolic diseases. Senescent cells instigate metabolic imbalances across various tissues, thereby fostering cellular senescence. This phenomenon creates a vicious cycle that precipitates the emergence of diverse metabolic diseases. However, the precise role of cellular senescence in metabolic diseases, including its regulatory mechanisms and key molecules, remains to be fully elucidated. Further in-depth exploration of these roles and mechanisms can facilitate the development of novel approaches for the prevention and treatment of metabolic diseases by leveraging the perspective of cellular senescence. This process would involve the identification of fresh targets for the removal of senescent cells in metabolic diseases, improving the therapeutic potential and application value of targeted senescent cell removal in metabolic diseases and generating valuable basic research data for the exploration of safe and effective therapeutic approaches for metabolic diseases, along with clinical treatment entry points.
